# Plasma endostatin and its association with new-onset acute kidney injury in critical care

**DOI:** 10.1186/s40560-025-00820-z

**Published:** 2025-09-02

**Authors:** Hazem Koozi, Jonas Engström, Anders Larsson, Martin Spångfors, Hans Friberg, Attila Frigyesi

**Affiliations:** 1 Department of Clinical Sciences, Anaesthesiology and Intensive Care, Lund University, SE-22185 Lund, Sweden; 2 Department of Anaesthesia and Intensive Care, Skåne University Hospital, SE-29133 Kristianstad, Sweden; 3 Department of Medical Sciences, Clinical Chemistry, Uppsala University, SE-75185 Uppsala, Sweden; 4 Department of Intensive and Perioperative Care, Skåne University Hospital, SE-20502 Malmö, Sweden; 5 Department of Intensive and Perioperative Care, Skåne University Hospital, SE-22185 Lund, Sweden

**Keywords:** Intensive care, Critical care, Endostatin, Acute kidney injury, Renal replacement therapy, Mortality.

## Abstract

**Background:**

Endostatin is a promising biomarker for predicting acute kidney injury (AKI) and mortality in the intensive care unit (ICU). We investigated plasma endostatin levels at ICU admission as predictors of new-onset AKI within 48 h after ICU admission, renal replacement therapy (RRT) within 7 days after ICU admission, and 30-day mortality.

**Methods:**

A retrospective multicentre study was performed with admissions to four ICUs. Blood samples were prospectively obtained at ICU admission and retrospectively analysed. The Kidney Disease: Improving Global Outcomes (KDIGO) criteria defined AKI. Endostatin at ICU admission was compared to and adjusted for creatinine, cystatin C, and the Simplified Acute Physiology Score 3 (SAPS-3). Regression models and mean areas under the receiver operating characteristic curves (AUCs) from repeated cross-validation were assessed.

**Results:**

In total, 4732 admissions were included. Endostatin was associated with new-onset AKI (OR 1.7, 95% CI 1.5$-$1.9), new-onset stage 3 AKI (OR 1.4, 95% CI 1.2$-$1.6), and RRT (OR 1.2, 95% CI 1.05$-$1.4) independently of creatinine, cystatin C, and SAPS-3. Endostatin was superior to creatinine and cystatin C in predicting new-onset AKI (mean AUC 0.67 vs. 0.63, *p* < 0.001). Adding endostatin to creatinine improved the prediction of new-onset AKI and new-onset stage 3 AKI, but not the need for RRT. Endostatin was not associated with 30-day mortality after adjusting for the SAPS-3 score.

**Conclusions:**

Endostatin at ICU admission is an independent predictor of new-onset AKI and may improve early AKI risk assessment in the ICU. However, its predictive value for RRT and 30-day mortality appears limited. External validation and studies on its clinical utility are warranted.

## Background

Acute kidney injury (AKI) is a frequent complication of critical illness, associated with substantial morbidity and mortality [1–3]. The clinically most established biomarker for AKI is creatinine. However, creatinine is a suboptimal indicator of AKI, as up to half of the glomerular filtration rate (GFR) may be lost before creatinine rises. The rise in creatinine is also relatively slow, especially in patients with an elevated baseline creatinine level [4]. Timely detection of AKI is crucial, as it facilitates prompt monitoring and can influence clinical decisions regarding hemodynamics, fluid management, and medication use. Novel biomarkers correlating with renal injury and/or function could play a vital role in this early detection [5].

Endostatin is a broad-spectrum angiogenesis inhibitor [6, 7]. It is a naturally occurring fragment of type XVIII collagen, an integral part of the basement membranes of renal tubular epithelium, Bowman’s capsule, mesangium, and renal capillaries [8]. Beyond endostatin’s roles in cancer biology, studies have demonstrated that it is involved in inflammatory and cardiovascular diseases [9–12]. Endostatin has shown prognostic value in pulmonary arterial hypertension, heart failure, and in a general elderly population [13–15]. Elevated plasma endostatin is also associated with chronic kidney disease (CKD) [16, 17].

Furthermore, endostatin has shown potential as a marker of disease severity and prognosis in intensive care. Several studies have reported associations between elevated endostatin levels and the risk of AKI, as well as renal recovery and mortality in AKI patients [18–21]. In COVID-19, endostatin has been associated with disease severity and AKI [22–24]. However, findings have been inconsistent [25]. Additionally, most studies evaluating endostatin in intensive care have been relatively small. The precise role and applicability of endostatin as a predictive biomarker in intensive care require further investigation in larger patient cohorts.

### Objectives

We aimed to assess plasma endostatin at ICU admission as an independent predictor of new-onset AKI, renal replacement therapy (RRT), and 30-day mortality in critically ill patients. Furthermore, we compared the predictive performance of endostatin with that of creatinine, cystatin C, and the Simplified Acute Physiology Score 3 (SAPS-3). We evaluated whether incorporating endostatin with these established markers improves prediction.

## Methods

### Study design and setting

A retrospective multicentre cohort study was performed as part of the SWECRIT project [26, 27]. The Strengthening the Reporting of Observational Studies in Epidemiology (STROBE) guidelines were followed [28].

Adult admissions to one of four general ICUs in southern Sweden (Skåne University Hospital in Lund and Malmö, Helsingborg Hospital, and Kristianstad Hospital) between 2015 and 2018 were consecutively included. Blood samples were prospectively obtained at ICU admission and preserved in the SWECRIT biobank for later retrospective analyses.

### Participants

All ICU admissions were screened for inclusion. Exclusion criteria included ICU discharge alive within 24 h, missing biobank samples, incorrect sampling or handling of biobank samples, patients or their next of kin opting out of the study, and ICU transfers without reobtained blood samples.

### Variables

Endostatin, creatinine, and cystatin C were analysed from blood samples obtained at ICU admission. Creatinine was also analysed on the first 2 days of the ICU stay or until ICU discharge, whichever occurred first. For baseline creatinine, the value closest to ICU admission within 7 to 365 days before was recorded. If baseline creatinine was missing, it was estimated using the 2021 Chronic Kidney Disease Epidemiology Collaboration (CKD-EPI) creatinine equation, assuming a baseline estimated glomerular filtration rate (eGFR) of 75 mL/min/1.73 $\textrm{m}^2$ [29]. CKD was defined as eGFR < 60 mL/min/1.73 $\textrm{m}^2$ [30]. Urine output was recorded on the two mornings following ICU admission or until ICU discharge. If urine output could not be monitored for 24 h, it was estimated by extrapolating from hourly urine output. AKI was defined as fulfilment of the Kidney Disease: Improving Global Outcomes (KDIGO) criteria within 48 h after ICU admission [30]. New-onset AKI was defined as the absence of AKI at ICU admission, followed by its subsequent development within 48 h after ICU admission. Admissions with AKI or missing AKI status at ICU admission were excluded from analyses of new-onset AKI. Similarly, new-onset stage 3 AKI was defined as the absence of stage 3 AKI at ICU admission, followed by its subsequent development within 48 h after ICU admission. For admissions to be classified as not having AKI at a particular time point, both creatinine and urine output data had to be available. For admissions to be classified as having AKI at a particular time point, missing creatinine or urine output data was accepted as long as they met KDIGO criteria. RRT was regarded as AKI unless the admission had a diagnosis of dialysable intoxication, in which case, they needed to fulfil another KDIGO criterion. For the outcome of RRT, we evaluated its initiation in the ICU within 7 days after ICU admission.

For variables included in SAPS-3 and the Sequential Organ Failure Assessment (SOFA) (excluding admission creatinine), the worst recorded values within 1 h of ICU admission were used [31, 32]. The value closest in time, within 24 h, of ICU admission was recorded for C-reactive protein (CRP) and lactate.

Immunosuppressive treatment, metastatic and haematological cancer, cirrhosis, and chronic heart failure were all defined according to SAPS-3 [31].

### Data sources

Endostatin, cystatin C, and admission creatinine were retrospectively batch analysed from prospectively collected blood samples. Blood samples were collected at ICU admission using ethylenediamine tetraacetic acid (EDTA) vacutainers and centrifuged to obtain EDTA plasma. The plasma samples were aliquoted and stored in the SWECRIT biobank at -80 $^\circ$ C. Samples had to be collected within 6 h of ICU admission. In cases where the sampling time was missing, samples were included if the freezing time fell within the 6-hour time frame.

Endostatin analyses were performed using commercial sandwich kits (DY1098, R&D Systems, Minneapolis, MN, USA). A monoclonal antibody specific for endostatin was coated onto microtiter plates. The plates were blocked with bovine serum albumin and then washed. Samples and standards were added to the wells, after which the peptide was bound to the immobilised antibodies. A biotinylated endostatin-specific antibody was added after the sample was washed. A streptavidin-HR conjugate was added to the wells after incubation and washing, and a substrate solution was added after another incubation and washing cycle. The absorbance was measured in a SpectraMax 250 (Molecular Devices, Sunnyvale, CA, USA). Endostatin values were determined by comparing the optical density of the samples to the standard curve. All assays were calibrated against highly purified recombinant human endostatin. Measurements were performed in a blinded manner, without knowledge of the clinical diagnoses. The total coefficient of variation for the endostatin assay was approximately 6%.

Admission creatinine and cystatin C were analysed on a Mindray BS380 chemistry analyser (Mindray Medical International, Shenzhen, China) using IDMS traceable enzymatic creatinine reagents from Abbott Laboratories (Abbott Park, IL, USA) and particle-enhanced turbidimetric cystatin C reagents from Gentian (Moss, Norway). Creatinine levels at baseline and on ICU days 1 and 2 were also analysed using the enzymatic method.

Body weight, body mass index, diabetes mellitus, hypertension, and chronic dialysis were manually collected from electronic medical records. All other clinical data were automatically extracted from electronic medical records.

### Study size

The sample size was determined by the number of ICU admissions during the study period, the ICU length of stay, the validity of blood samples, and the number of patients opting out.

### Bias

Treating clinicians and data collectors were unaware of endostatin levels, but they had access to creatinine and, occasionally, cystatin C levels as part of routine intensive care. Knowledge of creatinine levels may have influenced clinical decisions, including the initiation of RRT. Trained data collectors performed the manual data recording. Guidelines for data collection were standardised and precisely outlined. The management of missing data was collectively discussed and decided upon in the study group.

### Statistics

All statistical analyses were performed in R [33]. The median value was calculated for general characteristics, SAPS-3, physiological parameters, and biochemistry. The mean value was calculated for the Glasgow Coma Scale (GCS) and SOFA score. The Kruskal-Wallis test was used to compare medians. Pearson’s chi-squared test, or the chi-square test of independence, was used to compare proportions. Analysis of variance was used to compare means. Significance was determined as a *p*-value of less than 0.05.

In cases where both baseline creatinine and body weight were missing, body weight was imputed using linear regression based on age and sex, and the imputed value was subsequently used to estimate baseline creatinine for AKI classification.

Local polynomial regression was employed to show trends in the association between endostatin, outcomes, and creatinine [34].

Logistic regression was utilised to create prediction models of new-onset AKI, new-onset stage 3 AKI, RRT, and 30-day mortality as binary outcomes. To facilitate the interpretation and comparison of odds ratios (ORs), continuous predictor variables were standardised by z-normalisation. Biomarkers (endostatin, creatinine, and cystatin C) were log-transformed before z-normalisation due to skewed distributions. The SAPS-3 score was z-normalised without log transformation. For new-onset AKI, new-onset stage 3 AKI, and RRT, adjusted odds ratios (ORs) were estimated from a model including endostatin, creatinine, cystatin C, and SAPS-3 simultaneously. For 30-day mortality, the adjusted OR for endostatin was derived from a model that included both endostatin and SAPS-3.

Model performance was evaluated using 10-fold cross-validation repeated 20 times. Discrimination was assessed using the mean area under the receiver operating characteristic (ROC) curve (AUC), with 95% confidence intervals (CI) derived from the cross-validation resamples. Differences in AUC between models were evaluated using the method proposed by DeLong et al. [35].

## Results

### Participants

**Fig. 1:**
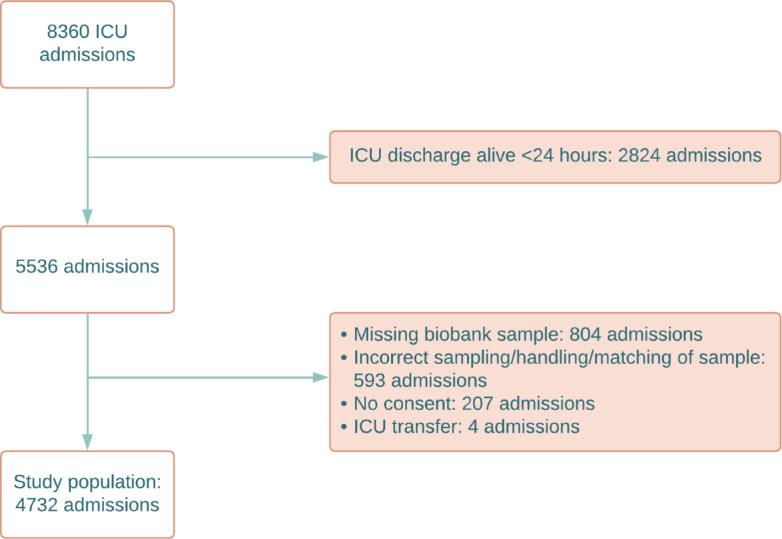
Flow chart of included and excluded ICU admissions. *ICU* Intensive Care Unit

A total of 8360 ICU admissions were identified. After exclusions, the study population consisted of 4732 ICU admissions. See Fig. 1. A total of 321 admissions (6.8%) were ICU readmissions.

### Descriptive data

The median age was 68 years (interquartile range [IQR] 56–75 years), and males comprised 60% of the study population. The rate of CKD was 17%. The median SAPS-3 score was 64 (IQR 52–75). The mean SOFA score was 6.9 (standard deviation [SD] 4.0). The median baseline creatinine was 79 $\upmu$mol/L (IQR 62–110 $\upmu$mol/L), whereas the median creatinine at ICU admission was 100 $\upmu$mol/L (IQR 69–160 $\upmu$mol/L). Baseline creatinine levels were missing in 1781 admissions (38%). The median cystatin C was 1.2 mg/L (IQR 0.77$-$2.0 mg/L). The median endostatin was 63 ng/mL (IQR 45–90 ng/mL). The three most common primary diagnoses were cardiac arrest (10%), septic shock (9.9%), and respiratory failure (5.3%).

Admissions with higher endostatin were older, had higher body mass index, higher rates of most comorbidities, higher SAPS-3 and SOFA scores, and higher degrees of organ dysfunction, except for GCS. They also had higher creatinine, cystatin C, lactate, and CRP levels, as well as lower platelet counts. See Table 1.

**Table 1:** Characteristics of the study population divided into plasma endostatin tertiles

Endostatin (ng/mL)	< 51	51–78	> 78	*p*-value	Missing (%)
n (%)	1590 (34)	1559 (33)	1559 (33)		0
Age (years)	61 (45–71)	70 (60–77)	71 (63–78)	< 0.001	0
Male sex (%)	61	60	60	0.61	0
Body mass index (kg/m^2^)	25 (22–30)	27 (23–30)	27 (24–32)	< 0.001	70
Comorbidities					
Immunosuppressive therapy (%)	6.6	9.0	9.6	0.0061	0
Metastatic cancer (%)	11	12	11	0.34	0
Haematological cancer (%)	2.0	3.3	3.1	0.067	0
Cirrhosis (%)	2.7	1.5	2.0	0.053	0
Chronic heart failure (%)	4.4	9.2	18	< 0.001	0
Hypertension (%)	13	21	23	< 0.001	46
Diabetes mellitus (%)	4.8	11	16	< 0.001	58
Chronic kidney disease (%)	4.2	11	36	< 0.001	38
Chronic dialysis (%)	0.13	0.25	3.0	< 0.001	58
Illness severity					
SAPS-3 score	56 (45–67)	64 (53–74)	71 (60–81)	< 0.001	0.042
SOFA score^*^	5.7 (3.6)	7.1 (3.9)	8.7 (3.9)	< 0.001	0.042
PaO_2_/FiO_2_ (kPa)	33 (19–49)	26 (16–40)	25 (16–41)	< 0.001	14
Mean arterial pressure (mmHg)	68 (60–80)	65 (58–78)	64 (50–73)	< 0.001	2.9
Vasopressor therapy (%)	46	54	56	< 0.001	6.2
Glasgow Coma Scale^*^	12 (4.4)	11 (4.4)	11 (4.2)	0.80	7.5
Laboratory values					
Baseline creatinine ($\upmu$mol/L)	69 (56–82)	76 (60–94)	108 (77–164)	< 0.001	38
Creatinine ($\upmu$mol/L)	74 (57–96)	96 (70–132)	170 (115–290)	< 0.001	0
Cystatin C (mg/L)	0.76 (0.59–1.1)	1.2 (0.84–1.6)	2.2 (1.5–3.1)	< 0.001	0
Platelet count (x10$^9$/L)	200 (150–270)	220 (150–300)	220 (140–280)	0.0010	5.6
Lactate (mmol/L)	1.8 (1.1–3.3)	2.1 (1.2–4.1)	2.4 (1.2–5.3)	< 0.001	0.063
C-reactive protein (mg/L)	17 (2.4–94)	43 (7.2–140)	77 (21–200)	< 0.001	0.51
Bilirubin ($\upmu$mol/L)	10 (6.0–16)	10 (6.0–17)	10 (6.0–18)	0.14	6.3
Outcomes					
AKI within 48 h in ICU (%)	38	61	86	< 0.001	4.8
Stage 3 AKI within 48 h in ICU (%)	9.1	19	50	< 0.001	6.4
AKI on ICU admission (%)	18	37	66	< 0.001	0
AKI on ICU day 1 (%)	27	47	74	< 0.001	9.2
AKI on ICU day 2 (%)	21	34	57	< 0.001	31
RRT within 7 days in ICU (%)	2.5	8.2	26	< 0.001	0.34
ICU length of stay (days)	2.1 (1.5–4.1)	2.6 (1.5–5.0)	2.8 (1.6–5.2)	< 0.001	0.042
30-day mortality (%)	21	31	40	< 0.001	0.021

Variables are at ICU admission unless otherwise specified. Values are medians with interquartile ranges unless otherwise specified. *P*-values were calculated using the Kruskal-Wallis test and the chi-square test of independence as appropriate, unless otherwise specified. ^*^Presented as means with standard deviations, and p-value calculated using analysis of variance. *SAPS-3* Simplified Acute Physiology Score 3, *SOFA* Sequential Organ Failure Assessment,* PaO*_2_ Arterial Partial Pressure of Oxygen, *FiO*_2_ Fraction of Inspired Oxygen (%), *AKI* Acute Kidney Injury, *RRT* Renal Replacement Therapy, *ICU* Intensive Care Unit

### Outcomes

The rate of AKI was 62%, whereas the rate of new-onset AKI was 22%. The rate of stage 3 AKI was 26%, whereas the rate of new-onset stage 3 AKI was 16%. Of those with AKI, 35% had AKI stage 1, 23% had stage 2, and 42% had stage 3. The rate of RRT during the ICU stay was 14%, whereas the rate of RRT within 7 days after ICU admission was 12%. The median ICU length of stay was 2.5 days (IQR 1.5$-$4.8 days). Mortality within 30 days was 30%.

### Main results

**Fig. 2:**
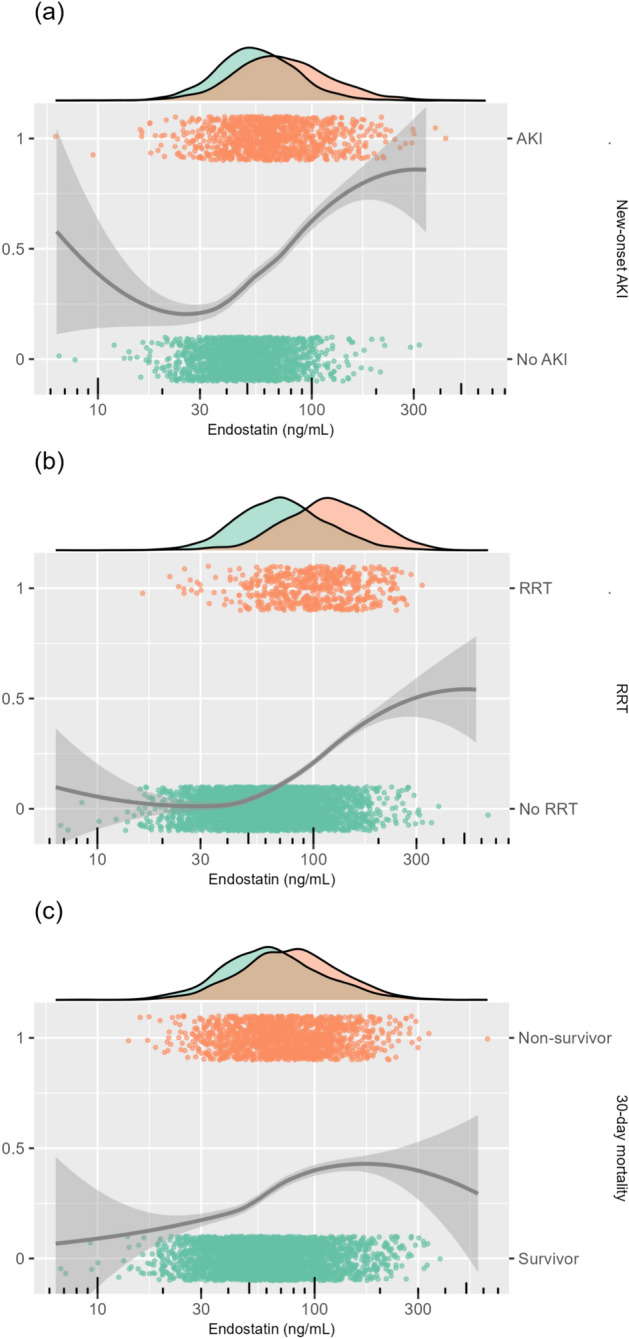
The relationship between plasma endostatin at ICU admission and new-onset AKI (**a**), RRT (**b**), and 30-day mortality (**c**) visualised in scatter and density plots. The x-axes have logarithmic scales. Each point represents an individual admission, showing the outcome (AKI, RRT, or 30-day mortality) in relation to endostatin level. Colours indicate outcome groups: green for admissions without the outcome and orange for admissions with the outcome. Continuous lines represent smoothed conditional means, illustrating the overall trend in outcome risk as endostatin levels increase. The accompanying shading indicates the corresponding 95% confidence intervals. The density plots above each panel illustrate the distribution of endostatin levels in patients with and without each outcome. *AKI* Acute Kidney Injury, *RRT* Renal Replacement Therapy, *ICU* Intensive Care Unit

**Fig. 3:**
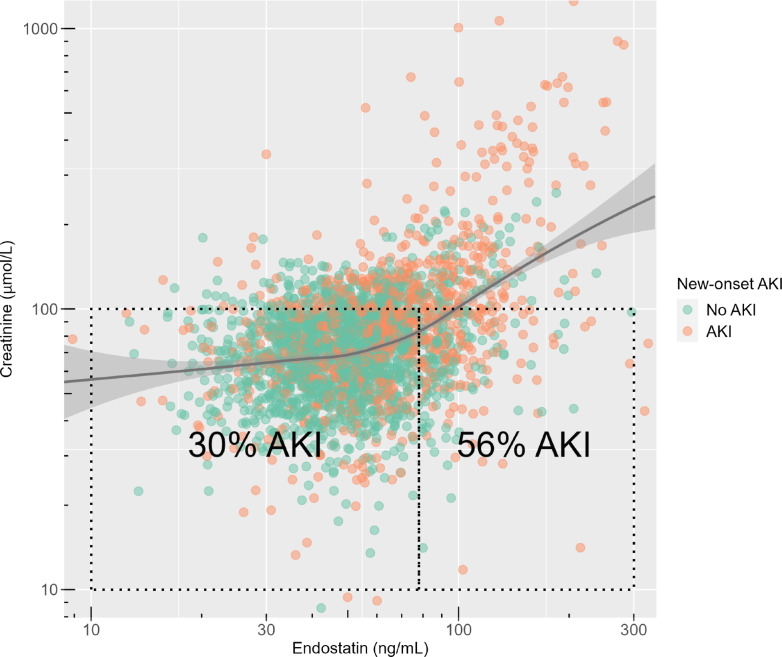
The relationship between plasma endostatin and creatinine at ICU admission and new-onset AKI visualised in a scatter plot. Each point represents an individual admission, illustrating the relationship between endostatin level and creatinine level. Colours indicate new-onset AKI status: green for admissions without AKI and orange for those with AKI. The box to the left indicates ICU admissions with creatinine 10–100 $\upmu$mol/L and endostatin 10–78 ng/mL, while the right box indicates creatinine 10–100 $\upmu$mol/L and endostatin 78–300 ng/mL. *P* < 0.001 for AKI rate comparison between the two boxes. The y- and x-axes have logarithmic scales. Continuous lines show smoothed conditional means, illustrating the overall trend in creatinine levels across increasing endostatin levels. The accompanying shading indicates the corresponding 95% confidence intervals. *AKI* Acute Kidney Injury, *ICU* Intensive Care Unit

**Fig. 4:**
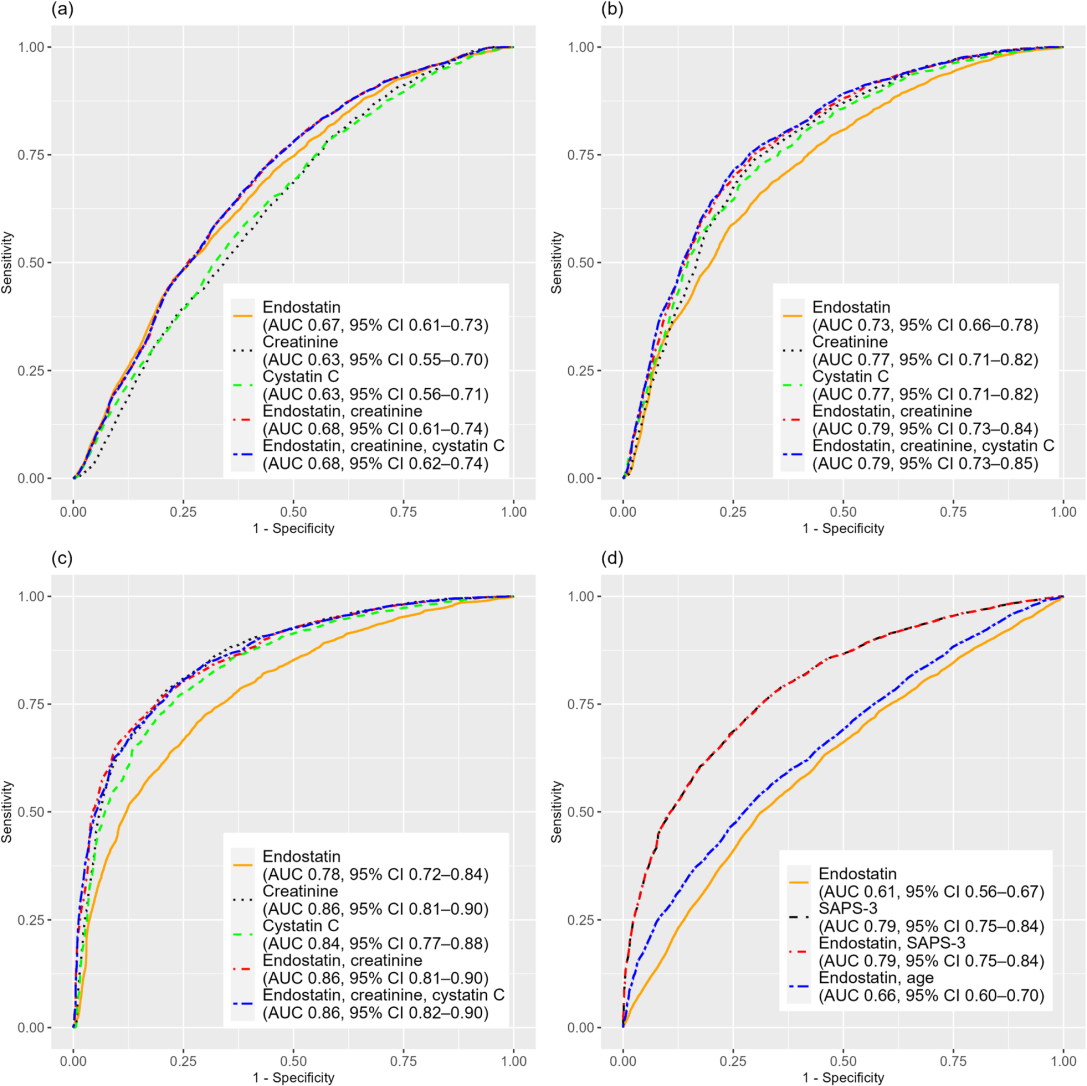
Receiver operating characteristic (ROC) curves and mean AUCs for endostatin and other variables at ICU admission for the prediction of new-onset AKI (**a**), new-onset stage 3 AKI (**b**), RRT (**c**), and 30-day mortality (**d**). *AUC* Area Under the Curve, *CI* Confidence Interval, *ICU* Intensive Care Unit, *AKI* Acute Kidney Injury, *RRT* Renal Replacement Therapy, *SAPS-3* Simplified Acute Physiology Score 3

The relationships between endostatin and new-onset AKI, RRT, and 30-day mortality are illustrated in Fig. 2. Higher endostatin levels were associated with an increased risk of all three outcomes. Admissions who developed new-onset AKI, required RRT, or died within 30 days had higher endostatin levels. The relationship between endostatin, creatinine, and new-onset AKI is displayed in Fig. 3. Higher endostatin was associated with elevated creatinine at ICU admission and an increased risk of new-onset AKI. Among patients with similar creatinine levels (10–100 $\upmu$mol/L), those with higher endostatin concentrations (78–300 ng/mL) had a greater proportion of new-onset AKI compared to those with lower endostatin (10–78 ng/mL; *p* < 0.001). ROC curves and AUCs of endostatin, creatinine, cystatin C, and SAPS-3 for predicting new-onset AKI, new-onset stage 3 AKI, RRT, and 30-day mortality are presented in Fig. 4.

#### New-onset AKI

Among 2601 admissions eligible for analysis, adjusted ORs were 1.7 (95% CI 1.5$-$1.9) for endostatin, 1.4 (95% CI 1.2$-$1.6) for creatinine, 1.0 (95% CI 0.88$-$1.2) for cystatin C, and 1.5 (95% CI 1.4$-$1.7) for SAPS-3. Endostatin had superior discrimination compared to creatinine (mean AUC 0.67 vs. 0.63, *p* < 0.001) and cystatin C (mean AUC 0.63, *p* < 0.001). Adding endostatin to creatinine improved mean AUC compared to creatinine alone (0.68 vs. 0.63, *p* < 0.001).

#### New-onset stage 3 AKI

Among 3942 admissions eligible for analysis, adjusted ORs were 1.4 (95% CI 1.2$-$1.6) for endostatin, 2.0 (95% CI 1.6$-$2.4) for creatinine, 1.5 (95% CI 1.3$-$1.8) for cystatin C, and 1.66 (95% CI 1.5$-$1.8) for SAPS-3. Endostatin had inferior discrimination to creatinine (mean AUC 0.73 vs. 0.77, *p* < 0.001) and cystatin C (mean AUC 0.73 vs. 0.77, *p* < 0.001). Adding endostatin to creatinine improved mean AUC (0.79 vs. 0.77, *p* < 0.001) compared to creatinine alone.

#### RRT

Among 4714 admissions eligible for analysis, adjusted ORs were 1.2 (95% CI 1.05$-$1.4) for endostatin, 2.7 (95% CI 2.3$-$3.2) for creatinine, 1.4 (95% CI 1.1$-$1.7) for cystatin C, and 1.5 (95% CI 1.3$-$1.6) for SAPS-3. Endostatin’s discrimination was inferior to creatinine (mean AUC 0.78 vs. 0.86, *p* < 0.001) and cystatin C (mean AUC 0.78 vs. 0.84, *p* < 0.001). Adding endostatin to creatinine did not enhance prediction (mean AUC 0.86 vs. 0.86, *p* = 0.10).

#### 30-day mortality

Among 4729 admissions eligible for analysis, the adjusted OR was 1.1 (95% CI 0.98$-$1.1) for endostatin. Endostatin was inferior to SAPS-3 (mean AUC 0.61 vs. 0.79, *p* < 0.001). Adding endostatin to SAPS-3 did not improve mean AUC (0.79 vs. 0.79, *p* = 1.0). Combining endostatin with age was also inferior to SAPS-3 (mean AUC 0.66 vs. 0.78, *p* < 0.001).

## Discussion

In this large ICU cohort, endostatin at ICU admission was independently associated with new-onset AKI, stage 3 AKI, and the need for RRT, even after adjustment for creatinine, cystatin C, and SAPS-3. Endostatin demonstrated superior discrimination compared to creatinine and cystatin C for predicting new-onset AKI, and improved predictive performance when added to creatinine for both new-onset AKI and stage 3 AKI. However, overall discriminatory performance was modest, and the absolute increase in AUC for stage 3 AKI was relatively small. In contrast, endostatin showed inferior performance to creatinine for predicting RRT and did not improve prediction when combined with creatinine, suggesting more limited utility for this outcome. Our results also show that endostatin, both alone and in combination with age, is inferior to SAPS-3 in predicting 30-day mortality. Adding endostatin to SAPS-3 did not improve discrimination. This makes endostatin unlikely to be a valuable predictor of mortality, but does not exclude the possibility that endostatin could be useful for predicting mortality in combination with other variables. Higher endostatin was associated with several comorbidities, organ dysfunction, ICU length of stay, and 30-day mortality.

In some smaller studies, endostatin has been shown to predict AKI in intensive care. A 2016 study in a similar setting found that adding endostatin to a predictive model significantly improved the prediction of AKI [18]. A more recent study concluded that admission endostatin, age, and creatinine predicted AKI and the need for RRT in a general ICU population [19]. Furthermore, they found that the combination of creatinine and endostatin was closely associated with the development of AKI.

However, a prospective multicentre study with 1112 patients from 2017 concluded that endostatin has limited value for predicting AKI and RRT, despite increasing endostatin with AKI stages. They found a slightly lower AUC for AKI and a considerably lower AUC for RRT. These contrasting results compared to our study could have several explanations. Our study had a larger sample size, a higher rate of AKI, a higher rate of RRT, and higher median endostatin values. They had a 90-day mortality rate of 19.8%, compared to a 30-day mortality rate of 30% in this study. There was also a difference in the AKI observation period compared to our study, and no direct comparison to creatinine. These differences in patient populations and methodology likely explain the discrepancies in results and conclusions between the studies. Endostatin may have greater predictive value in populations with a higher pretest probability of AKI. Despite the mentioned differences in patient populations, the AUC for 90-day mortality was comparable to our AUC for 30-day mortality [25]. This strengthens the conclusion that endostatin is unlikely to be of value in mortality prediction despite some conflicting results in relatively smaller studies [19].

The independent association between endostatin and new-onset AKI, stage 3 AKI, and RRT, despite adjustment for SAPS-3, suggests that it reflects a pathophysiological process beyond general illness severity. Endostatin elevation in AKI may be due to accelerated turnover of collagen XVIII, an integral part of the basement membranes in the kidney [8]. In animal AKI models, upregulated renal endostatin expression has been shown to precede deteriorating kidney function by several hours [36, 37]. However, since collagen XVIII is part of the basement membranes in many body structures, endostatin is unlikely to be specific to kidney injury [38]. It is also possible that endostatin reflects GFR, as its size (20kDa) should allow it to be relatively freely eliminated by glomerular filtration [6, 39, 40].

While the role of cystatin C in AKI remains debated, it has previously shown strong predictive performance [41]. In our study, its performance for predicting new-onset AKI was more modest, possibly due to the exclusion of patients with AKI at admission, who may have had higher cystatin C levels. Although endostatin outperformed cystatin C for new-onset AKI overall, cystatin C was superior for predicting new-onset stage 3 AKI and RRT. As a GFR marker, cystatin C may reflect more advanced renal impairment, while endostatin may capture earlier injury that has not yet translated into reduced GFR. Additionally, as cystatin C was occasionally available in routine care, it may have influenced RRT decisions, introducing potential bias.

Fair comparisons between endostatin and creatinine are difficult since most AKI classifications rely heavily on creatinine, including the KDIGO criteria used in this study [30, 42]. This creates a potential bias in favour of creatinine when it is used both as a predictor and as part of the outcome definition. However, in this study, new-onset AKI was defined based on prospective changes in creatinine within 48 h after ICU admission, while only creatinine at admission was used as a predictor. Although elevated or rising creatinine alone is not sufficient to indicate dialysis, it likely influences clinical decisions to initiate RRT, further complicating comparisons with other biomarkers.

Creatinine is associated with a delay in identifying renal function loss [4, 5]. Incorporating biomarkers such as endostatin into clinical practice could enhance early risk assessment and facilitate prompt identification of AKI, which is associated with substantial mortality and morbidity [1, 3]. Timely recognition of AKI is crucial for mitigating hypoperfusion, nephrotoxic medications, and other associated risk factors. Early AKI-risk stratification using biomarkers like endostatin could guide future clinical trials and identify subgroups that may benefit from interventions related to haemodynamics and novel pharmacological treatments that have previously failed to show clear benefit. Biomarker-based risk stratification may pave the way for more personalised and proactive management strategies to prevent progression of AKI, thereby improving patient outcomes.

This is the most extensive study on endostatin in an ICU setting to date. It was conducted across four ICUs of varying size and case mix, which may support generalisability within similar healthcare systems. We used a comprehensive approach to data collection, including prospectively collected blood samples, electronic medical records, and registry data. Multivariable analyses were adjusted for both established markers of renal function and illness severity, and model performance was internally validated using repeated cross-validation.

While we utilised robust methodologies to mitigate biases, the retrospective design of this study was still subject to limitations such as incomplete data. The study was conducted in ICUs within a single geographic region, which may limit generalisability. Comorbidities not included in SAPS-3 and baseline creatinine had considerable rates of missing data. Body weight was also missing in many patients and needed to be imputed for baseline creatinine estimation. Missing or inaccurate baseline creatinine could lead to an overestimation of AKI at ICU admission, as CKD might be misclassified as AKI. However, the outcome of new-onset AKI should not have been affected to the same extent by such bias.

In our previous work, assessing endostatin in critically ill patients with COVID-19, we analysed endostatin as a categorical variable. This was suitable as risk increased with endostatin levels up to approximately 200 ng/mL, followed by a decline, forming a U-shaped relationship. Other cut-offs were predefined based on prior evidence and pragmatic reasoning [24]. The choice to analyse endostatin as a continuous variable in the present study was based on poorly defined baseline or normal endostatin levels and the absence of a clear U-shaped relationship between endostatin and outcomes. We presented endostatin tertiles for descriptive purposes, allowing for comparison across endostatin levels without presupposing threshold effects.

We excluded patients discharged alive within 24 h to avoid including low-risk admissions that were primarily admitted for observation. While this limits generalisability to lower-risk ICU populations, it aligns with our focus on evaluating early AKI risk in higher-risk patients, where timely identification may have the most significant clinical impact. ICU admissions, rather than individual patients, were analysed to reflect the dynamic and rapidly changing nature of AKI risk between admissions. While this approach may introduce clustering and affect standard error estimation, the readmission rate was relatively low, and we considered each ICU stay to represent a distinct clinical episode. Nonetheless, we acknowledge the potential impact of non-independence.

External validation of endostatin in predicting AKI and RRT is warranted. Future studies should also explore the clinical applicability of endostatin, including the definition of clinically relevant thresholds and its integration with other emerging AKI biomarkers, to further improve early AKI risk stratification. Lastly, our study focused on endostatin at the time of ICU admission, providing a snapshot of its predictive value. Studies incorporating serial assessments of endostatin may provide further insights into its prognostic utility.

## Conclusions

This extensive multicentre study demonstrates that endostatin at ICU admission is an early independent predictor of new-onset AKI and stage 3 AKI. Endostatin outperformed both creatinine and cystatin C in predicting new-onset AKI, and improved predictive performance when added to creatinine for both new-onset AKI and stage 3 AKI. However, overall discriminatory performance was modest. Although independently associated with the need for RRT, endostatin did not provide incremental value for RRT prediction. Furthermore, it showed a limited association with 30-day mortality and did not improve predictive performance when added to SAPS-3. These findings suggest that endostatin may enhance early risk assessment for AKI at ICU admission, while its utility for predicting RRT and mortality appears to be limited. Future studies should validate these findings in external cohorts and further assess the clinical utility of endostatin.

## Data Availability

The datasets generated and analysed during the current study are not publicly available due to limitations in the ethical approval of the study and data management policies of Region Skåne. However, they are available from the corresponding author on request.

## Abbreviations

AKI: Acute kidney injury
AUC: Area under the curve
CCI: Charlson comorbidity index
CI: Confidence interval
CKD: Chronic kidney disease
CKD-EPI: Chronic kidney disease epidemiology collaboration
CRP: C-Reactive protein
EDTA: Ethylenediamine tetraacetic acid
eGFR: Estimated glomerular filtration rate
GFR: Glomerular filtration rate
GCS: Glasgow coma scale
ICU: Intensive care unit
IQR: Interquartile range
KDIGO: Kidney disease: improving global outcomes
OR: Odds ratio
ROC: Receiver operating characteristic
RRT: Renal replacement therapy
SAPS-3: Simplified acute physiology score 3
SIR: Swedish intensive care registry
SOFA: Sequential organ failure assessment
SD: Standard deviation
STROBE: Strengthening the reporting of observational studies in epidemiology
